# Overexpression of the Pdx-1 Homeodomain Transcription Factor Impairs Glucose Metabolism in Cultured Rat Hepatocytes

**DOI:** 10.3390/molecules13102659

**Published:** 2008-10-28

**Authors:** Rudolf Tito Pillich, Gianfranco Scarsella, Gianfranco Risuleo

**Affiliations:** 1 Dipartimento di Biologia Cellulare e dello Sviluppo, Sapienza Università di Roma, P.le Aldo Moro, 5 – 00185 Roma, Italy; E-mail: rpillich@ucsd.edu (R-T. P.), gianfranco.scarsella@uniroma1.it (G. S.); 2 Dipartimento di Genetica e Biologia Molecolare, Sapienza Università di Roma, P.le Aldo Moro, 5 – 00185 Roma, Italy

**Keywords:** Pdx-1, Hexokinase-2, Transcriptional promoters, Glucose metabolism

## Abstract

The Pdx-1 transcription factor plays crucial functions both during pancreas development and in the adult β cells. Previous studies have indicated that ectopic Pdx-1 expression in liver or intestinal primary and immortalized cells is sufficient to promote activation of insulin gene expression. This work is focused on the molecular and physiological consequences of Pdx-1 overexpression in liver cells. We present evidence that Pdx-1 affects the level of expression of one of the four mammalian hexokinase isozymes. These are glucose phosphorylating enzymes involved in essential cellular functions such as glucose sensing, metabolic energy production and apoptosis. Specifically, our data show that over-expression of Pdx-1 in cultured hepatocytes is able to repress the expression of hexokinase 2 (Hxk 2) and the phenomenon is mediated via binding of Pdx-1 to a specific sequence on the Hxk 2 gene promoter. As a consequence, liver cells over-expressing Pdx-1 present interesting alterations concerning glucose metabolism.

## Introduction

Glucose is the main source of energy in eukaryotes and constitutes the basis for the production of ATP. Plants can synthesize their own glucose, while animals obtain it from food. Due to defined circadian cycles, animals regulate the availability of this sugar in all cells at any given time. Under normal metabolic conditions, the concentration of glucose in the blood is kept constant; therefore different enzymes and carrier-molecules are involved in the delivery of this sugar to the cell. Specific carriers, belonging to two different super-families are involved in these processes: the SGLT family, formed by three different members of active transporters (or symporters), and the GLUT family, which includes 12 members of facilitator carriers. The first one represents the intestinal and most important Na^+^-dependent glucose transporter and is responsible for its uptake at microvilli level [1,2,3]. The affinity for glucose is actually different among the GLUT proteins; furthermore the expression of these genes is regulated both by the glucose level and the interactions with other gene family products: the hexokinases. When glucose blood level decreases, sugar homeostasis ensures glucose production from non saccharidic compounds such as amino-acids, Krebs cycle intermediates, lactate and glycerol. On the contrary, when the glucose level increases liver cells transform it into glycogen, an insoluble polymer that can be easily stored in the cytoplasm. The 6-phospho-glucose, however, can enter several different pathways. Therefore, selective expression of different hexokinase isoforms, with different catalytic and regulatory properties, is likely to be an important factor in determining the pattern of glucose metabolism. Four hexokinase isozymes are known and these are generally referred to as Type I, II, III and IV, the latter being also known as glucokinase (GCK). The Types I-III are 100 kDa molecules, while GCK is a 50 kDa molecule, which has a high degree of similarity with the other isozymes particularly at the N and C-terminal domains; in addition, this enzymes have a conserved structure among a wide variety of organisms [4]. It is widely accepted that differences in sub-cellular localization of the isozymes may result in compartmentalization of glucose metabolism with “channelling” of 6-phospho-glucose to particular metabolic pathways [5, 6]. Another key role in the metabolism of glucose is played by the PDX-1 protein. This polypeptide is a 283 amino-acid homeodomain transcription factor that binds to specific elements in the mammalian genome and, cooperating with other ubiquitous or tissue specific transcription factor, drives the expression of many different genes, the most important being insulin [7,8,9,10,11,12,13,14,15]. PDX-1 has a predicted molecular weight of 31 kDa, although the functional form of the protein presents a number of different post-translational modifications that specifically confer unique features to this molecule. It is in fact well established that glucose and a number of other nutrients as well as some hormones, regulate Pdx-1 at several different levels: glucose-stimulated PDX-1 binding to A box elements occurs rapidly and involves phosphorylation of the transcription factor [16,17,18]. The signalling pathway by which glucose stimulates PDX-1 binding to DNA involves PI3-K and the stress activated p38SAPK, however, it is not yet clear how glucose activates the PI3-K pathway [19]. In addition to its effect on the phosphorylation of PDX-1, its DNA binding activity and its intracellular distribution, glucose also directly stimulates the trans-activation potential of the NH_2_-terminal activation domain by an undefined mechanism [20]. However, a number of hormones such as GLP-1 and TGFβ have effects on Pdx-1 either at the level of DNA binding activity, trans-activation potential or gene expression [21, 22]. Many reports indicate that over-expression of Pdx-1 can drive insulin gene expression in the liver [23,24,25], therefore we focused our attention on the role of Pdx-1 over-expression in liver cells; particularly, we examined the possibility that Pdx-1 over-expression could stimulate insulin production in a differentiated normal rat hepatocyte cell line and then tried to evaluate other important metabolic consequences due to its over-expression. 

## Results and Discussion

### Over-expression of the Pdx-1 gene in cultured Clone 9 rat hepatocytes.

**Figure 1:**
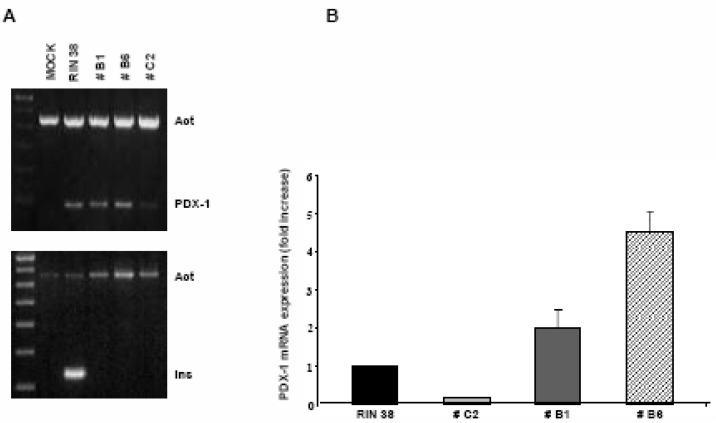
**A**) After transfection with construct pcDNA3/Pdx-1 and stable clone selection, RT-PCR was performed on total RNA. Note that all three clones analyzed, B1, B6 and C2, express the Pdx-1 gene at different levels but they do not express the insulin gene. Total RNA extracted from RIN 1046-38 cells was used as reference sample. **B**) The bar graph shows the result of Q-rtPCR analysis of Pdx-1 expression: clone C2 is expressing Pdx-1 at a very low level whereas clone B6 over-expresses the transcription factor. Results were normalized *vs* the Pdx-1 expression level detected in RIN38 cells (mean ± SEM).

We investigated whether Pdx-1 expression in liver cells is *per se* sufficient to trigger insulin production in our experimental model. To this end, an expression vector harboring the full length mouse Pdx-1 cDNA, was used to generate stable transfectants. Among the obtained clones, three were analyzed showing different levels in the expression of the Pdx-1 mRNA, namely C2, B1 and B6 (Figure 1).

**Figure 2:**
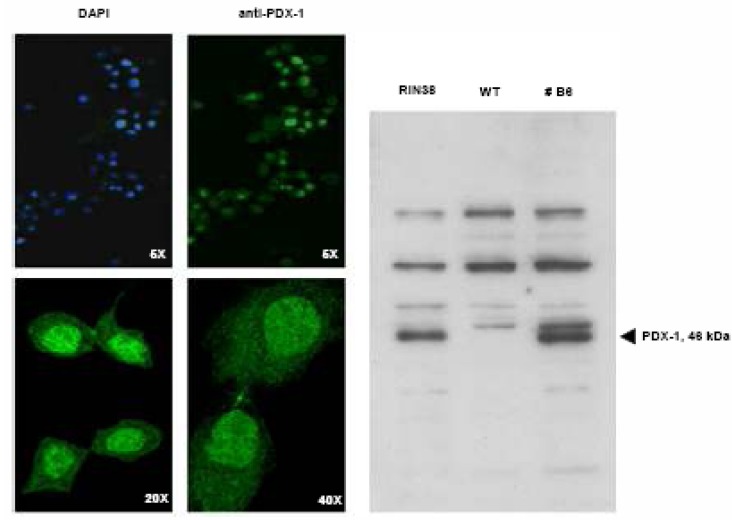
After transfection and selection of stable clones, the correct subcellular localization of the PDX-1 transcription factor was verified both by immunofluorescence analisys (left panels), and by Western blotting analysis on enriched nuclear extracts (right panel). Blue: nuclear DAPI staining. Green: PDX-1 staining. The two bottom immunofluorescence panels are higher magnification fields (20X and 40X) not related to the field shown in the top panels.

The cDNA obtained from RIN38 rat insulinoma cells was used as positive control. After conventional RT-PCR, the insulin mRNA was not detected in any of the analyzed clones, regardless of their different levels of Pdx-1 expression, as determined by quantitative real time PCR (Q-rtPCR). As over-expression can often also cause mis-localization of a protein, we decided to visualize the correct PDX-1 subcellular localization both by immunofluorescence and biochemical cell fractionation and immunoblotting. As shown in Figure 2, PDX-1 is mainly localized inside the nucleus even if a faint staining is also detectable in the cytoplasm. In the right panel, immunodetection of PDX-1 in nuclear extracts from RIN38 control cells, WT (Wild Type) and B6 hepatocytes is shown. The band has the correct molecular mass and is not present in the WT sample thus indicating specificity of the immunoreaction. Therefore, although Pdx-1 gene is expressed and the protein correctly localized within the cell, it is not sufficient to promote insulin expression in our cell model system.

### Modulation of the Hexokinase 2 (Hxk2) gene in Clone 9 hepatocytes over-expressing Pdx-1.

**Figure 3:**
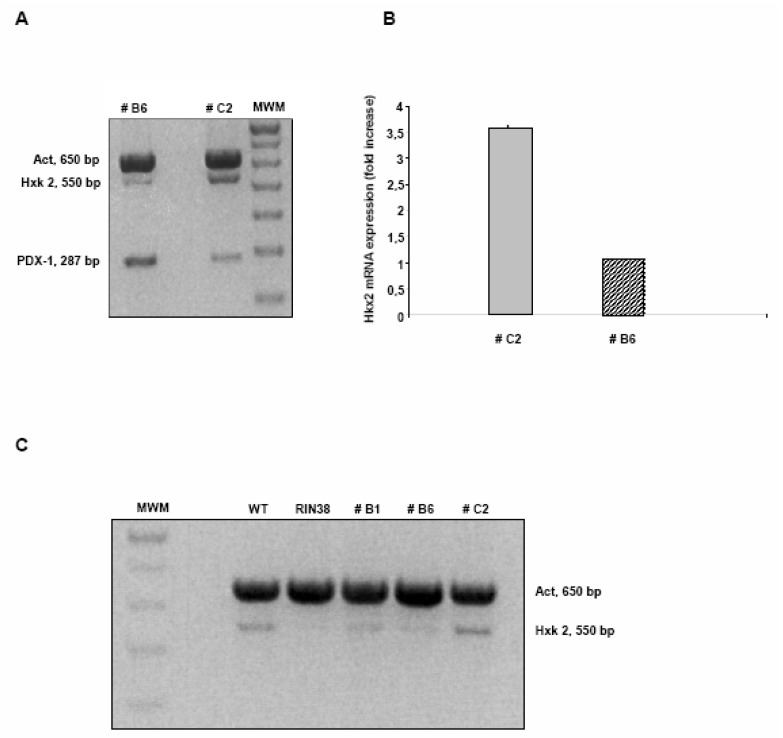
The stable expression of Pdx-1 in Clone 9 hepatocytes modulates the expression of the Hxk 2 gene. A) Multiplex RT-PCR shows that, the higher is the Pdx-1 expression, the lower is the Hxk 2 expression, and *viceversa*. B) The bar graph summarizes the results obtained after Q-rtPCR analysis: Hxk 2 expression is higher in the C2 clone and lower in the B6 clone, as already observed. The results were normalized *vs* the Hxk 2 gene expression level observed in B6 Clone 9 cells (mean ± SD). C) The Hxk 2 gene expression level is different in Rin38 cells, WT hepatocytes and Pdx-1 overexpressing clones B1, B6 and C2 as shown by duplex RT-PCR.

In light of these results we investigated the expression levels of different genes involved in glucose homeostasis and/or pancreatic development and function in hepatocytes over-expressing Pdx-1 (clone B6). The expression profile of the genes of interest (Table 1) was evaluated by quantitative real time PCR: among these genes, only the one coding for Hxk2 showed a significant alteration of the expression level. As already shown in Figure 1, B6 cells express Pdx-1 at a higher level as compared to C2 cells; on the contrary, the expression level of the Hxk 2 mRNA shows a completely opposite trend: in fact B6 cells exhibit a transcriptional level of Hxk 2 mRNA about four-fold lower than in C2 cells (Figure 3), while within the same cell population the mRNA for Pdx-1 is twenty-fold higher (compare to results in Figure 1). 

The opposite balance shown for the mRNA levels of both genes, indicate that Pdx-1 may act as a possible regulator for Hxk 2 gene expression.

**Table 1:** Primers used in cloning, PCR and EMSA experiments. The RT suffix indicates primers used for Q-rtPCR.

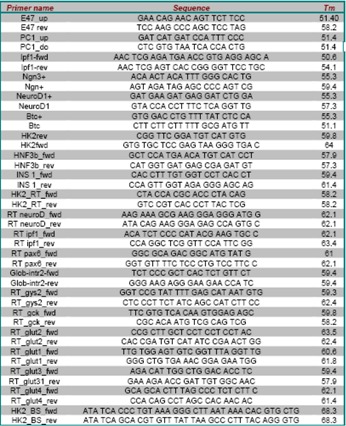

### PDX-1 binds to and regulates the Hxk 2 gene promoter.

**Figure 4:**
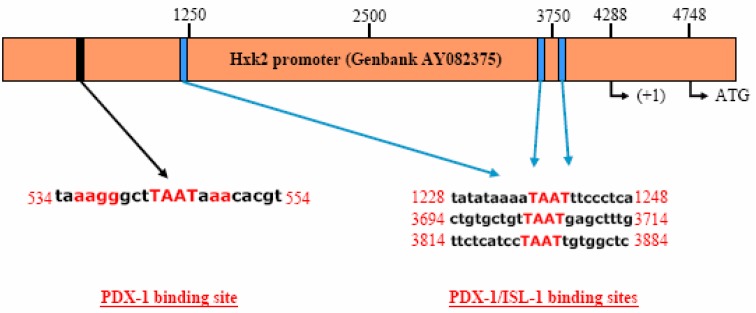
Schematic representation of the Hxk 2 gene promoter and predicted PDX-1 binding sites; the sequence between 534 and 554 has been used to generate the dsDNA probe for EMSA analysis. Letters in red color indicate the consensus sequence responsible for PDX-1 binding as well as the core TAAT module for general homeodomain transcription factor binding. The sequences shown in this figure were identified using the MatInspector software (www.genomatix.de).

Next, we analyzed the full length Hxk 2 promoter sequence for putative PDX-1 binding sites using an on-line prediction software (see Experimental Section). The software scored four putative PDX-1 binding sites (Figure 4), three of which were labeled as PDX-1/ISL-1 binding sites which contain only a core TAAT generic motif bound by any homeodomain transcription factor. A further putative binding sequence was identified in the distal promoter region. This sequence contains several conserved bases flanking the core TAAT motif and a deriving consensus sequence very similar to other known PDX-1 binding sequences. These are well characterized and are found in the promoters of genes regulated by PDX-1 including glucokinase and insulin genes. In light of this evidence we tested whether PDX-1 could regulate the Hxk 2 gene promoter activity operating on its regulatory sequences. Based on the identified consensus target, a double stranded DNA oligonucleotide probe was designed to be used in an *in vitro* Electrophoretic Mobility Shift Assay (EMSA).

As shown in Figure 5a, nuclear protein extracts from WT Clone 9 cells did not cause any molecular weight shift; conversely, nuclear extracts obtained from B6 cells produced an evident mobility retardation which was abolished when the binding reaction was carried out in the presence of a 100-fold specific cold competitor DNA. 

**Figure 5a:**
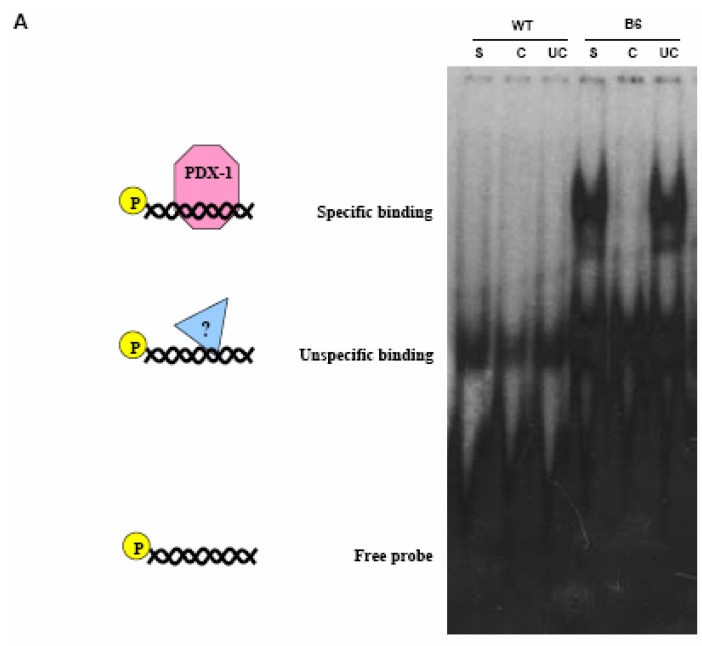
EMSA on nuclear extracts from WT and B6 Clone 9 cells using a radio-labeled dsDNA probe mimicking the distal region of the rat Hxk2 promoter and harboring the putative PDX-1 binding site identified with MatInspector (S, shift; C, competitor; UC, unspecific competitor).

**Figure 5b:**
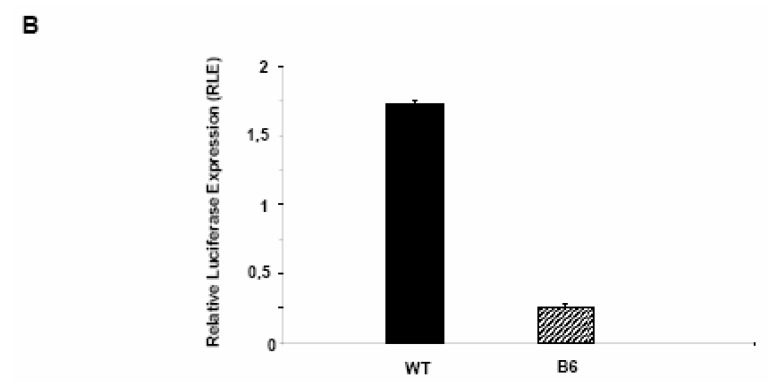
Expression of firefly luciferase driven by the Hkx2 promoter in WT and B6 Clone 9 hepatocytes: luciferase intensity is 6 fold higher in WT then B6 cells (mean ± SD).

As a control, cold unspecific competitor DNA was unable to abrogate the mobility shift. The result was further validated by an *in vivo* functional assay in which the full length Hxk 2 promoter region was used to direct firefly luciferase gene expression in both WT and B6 cultured Clone 9 hepatocytes. *Renilla* luciferase gene under the control of the constitutive Tk promoter was used as a normalizing reference factor. The expression levels of firefly luciferase driven by the Hxk 2 gene promoter appeared higher in WT rather than in B6 hepatocytes, as shown in Figure 5b, thus confirming our hypothesis that PDX-1 functions as a negative regulator of the Hxk 2 gene promoter activity.

### Glycogen synthesis is impaired in Clone 9 hepatocytes over-expressing Pdx-1.

**Figure 6:**
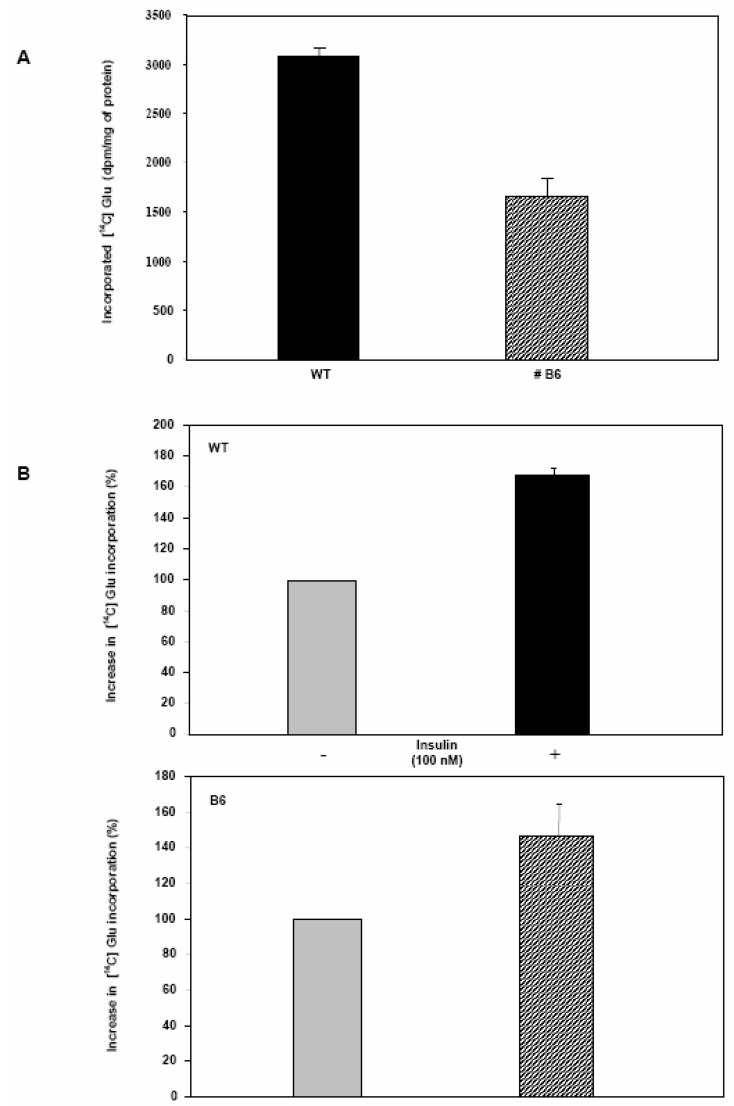
A) Glycogen synthesis is impaired in B6 cells as compared to WT Clone 9 cells, as shown by the reduced incorporation of [^14^C] Glucose. B) Insulin stimulation (1h at 37°) increases the percentage of [^14^C]-glucose incorporated both into in WT and B6 Clone 9 cells, even if at slightly different extents (mean ± SEM).

The isoform 2 of mammalian hexokinases has a high K_m_ value and therefore a low affinity for glucose; this enzyme can in fact bind and phosphorylate glucose when the intracellular sugar concentration is about 15 mM or higher. This feature indicates that Hxk 2 may phosphorylate glucose leading it towards the storage pathway in the form of glycogen [28]. Since PDX-1 significantly represses Hxk 2 gene expression, we thought that a parallel impairment in the glycogen synthesis rate could also occur. To this end, Clone 9 cell growth medium was supplemented with [^14^C] labeled glucose (see Material and Methods). The experiment was run in the presence and absence of insulin, both in WT and B6 cells and total glycogen was extracted to compare the rate of deposition (Figure 6a). The amount of radioactive glycogen is reduced by 50% in B6 cells as compared to WT cells. Furthermore, as expected, insulin administration increased the rate of glycogen synthesis in both cell lines almost to the same extent, indicating that the insulin sensing machinery and signaling pathway are not altered by Pdx-1 over-expression (Figure 6b). 

A number of specific functions have been described for the PDX-1 transcription factor in the last 10 years. In this work, we hypothesized that the forced over-expression of Pdx-1 in a non-pancreatic cell type could be a valid tool to uncover new functions. Recent works in fact indicated that Pdx-1 over-expression may be sufficient to stimulate insulin production in the liver, and that co-expression of other pancreatic markers (such as NeuroD1 and Betacellulin) can increase this effect and recover normo-glicemia in animals with SZT induced type I diabetes [23,24,25, 29]. Unfortunately, most of these reports failed to exclude that the correction of glycemia in SZT-dependent diabetic animal models could be explained by the regeneration of endogenous pancreatic beta-cells. 

While we were unable to detect insulin in hepatocytes over-expressing Pdx-1, we did nonetheless find out that this transcription factor imparts significant molecular and physiological changes in these non-pancreatic cells. The B6 clone over-expresses Pdx-1 and the protein has the correct sub-cellular localization, indicating that the increased presence of PDX-1 does not appear to be toxic. However the morphological observation of WT hepatocytes, shows that these cells are slightly smaller and elongated as compared to B6 cells. Also, WT cells exhibit dark granules of glycogen in the cytoplasm while these granules are less visible in B6 cells. These differences are readily detectable at a low confluence, but tend to disappear after establishment of the monolayer. A further phenotypic observation is that B6 cells show a diminished acidification of the culture medium with respect to WT cells, indicatinga lower catabolic activity (data not shown).

These phenotypic observations are supported by the results obtained with [^14^C]-labelled glucose: B6 hepatocytes incorporate less radioactive glucose into glycogen than WT cells, indicating that the anabolic activity is impaired in this cell type by the expression of Pdx-1. Thus, these observations suggest that once expressed in hepatocytes, Pdx-1 is able to regulate glucose metabolism, affecting an anabolic process such as glycogen synthesis. As a consequence, since glycogen synthesis and storage is one of the main functions of the hepatocyte, β cell has no need to synthesize glycogen: glucose must in fact be directed towards glycolysis, TCA cycle and oxidative phosphorylation in order to produce ATP. The increase of the ATP/ADP ratio will be the master signal triggering de-granulation and insulin release. Finely controlled release of insulin is indeed the main goal for a pancreatic β cell. Pdx-1-mediated modulation of glycogen synthesis is exerted via regulation of an important gene involved in glucose metabolism: the Hxk 2 gene. The promoter sequence of this gene contains several control regions bound by various different transcription factors: Pdx-1 binds one of these sequence motifs as demonstrated by our EMSA results, allowing the factor to partially down-regulate the expression of Hxk 2 gene. The PDX-1/ISL-1 dimer can bind three additional control regions, and these sequences may cooperatively increase the silencing of the Hxk 2 gene. This concept is supported by two observations: first, Hxk 2 is not completely repressed by PDX-1 because ISL-1 is not present in our hepatocyte model and therefore the three PDX-1/ISL-1 sites are not used. Secondly, in RIN38 cells the Hxk 2 gene is almost totally silent since its mRNA is barely detectable. Notably, these cells do express the ISL-1 transcription factor: as a result, in RIN38 cells the Hxk 2 gene expression can be repressed more efficiently both by PDX-1 alone and/or by the PDX-1/ISL-1 dimer. We would like to stress that the modulation of the Hxk 2 gene expression reflects the physiological role of the cell system: liver cells in fact need an active Hxk 2 gene to produce and accumulate glycogen; on the other hand, in pancreatic cells, the Hxk 2 gene is repressed, thus focusing the cell on ATP production and consequently on insulin secretion and neo-synthesis. The possibility that PDX-1 acts cooperatively with some other unidentified factors cannot of course be ruled out, one of these factors being ISL-1 in pancreatic β cells. 

The results obtained *in vitro* in the EMSA experiments, were confirmed by functional assays *in vivo*. In fact, using a quantitative reporter gene expression system, we were able to verify that gene expression from the Hxk 2 promoter was less efficient in B6 hepatocytes as compared to WT cells. The overall conclusion is that Pdx-1 over-expression in Clone 9 normal rat hepatocytes can efficiently reduce *de novo* glycogen synthesis by acting as a negative regulator on the Hxk 2 gene expression level, therefore causing a lower intra-cellular concentration of the enzyme. Acting on the rate of glycogen synthesis through the modulation of Hxk2 gene expression, PDX-1 could also exert “side effects” on other glucose-related energy production cellular processes such as glycogen mobilization and/or gluconeogenesis.

## Conclusions

In summary, our studies demonstrate that the PDX-1 transcription factor can significantly alter hepatocytes glucose metabolism by transcriptional regulation of at least one important gene of the glycolytic pathway. These results should therefore be taken into consideration when using PDX-1 as a key factor for approaches based on gene therapy. Hence, Pdx-1 over-expression is likely to affect negatively the metabolic function also in animal models. 

## Experimental Section

*Cell cultures, transfection and immunofluorescence:* Clone-9 rat hepatocytes (American Type Culture Collection, ATCC # CRL-1439) were maintained in DMEM-F12 (Gibco) supplemented with 10% fetal bovine serum, penicillin/streptomicin and Geneticin (100 μg/mL) when applicable. Cells were split every 3-4 days at a 1:10 ratio for not more than 10-15 times. Rat insulinoma RIN 1046-38 cells [26] were maintained in M-199 supplemented with 10% FBS, penicillin and streptomycin. Glucose was added to the final concentration of 11.1 mM. Cells were split every 4-5 days at a ratio of 1:5. For measurement of glycogen synthesis, cells were starved overnight in serum/glucose-free DMEM and then incubated in serum-free DMEM supplemented with 5.5 mM glucose, 1 μCi/mL (U-^14^C) glucose and with or without 100 nM insulin for 1 hour at 37°. Transient transfection experiments were performed using the Fugene 6 reagent from Roche according to the manufacturer’s instructions. Stable expressing clones were obtained using the CaCl_2_ transfection and serial dilution cloning methods. Immunofluorescence analysis was performed on 2% PFA fixed cell monolayer using a goat anti-PDX-1 antibody (see Antibodies and Western blotting section).

*Nucleic acids manipulation, constructs, RT-PCR and Q-rtPCR:* genomic DNA purification was performed using the DNeasy Tissue kit from while nucleic acids purification from agarose gel was performed using the Qiaquick gel extraction kit (Qiagen). Total RNA purification was achieved using the Nucleospin RNA extraction kit (Macherey-Nagel). The cDNA synthesis was performed using random primers, 1 μg of total RNA as template and 200 units of MMLV reverse transcriptase (Invitrogen). PCR were performed using a Geneamp 2400 (Applied Biosystems), and ExTaq DNA polymerase (Takara), while Q-rtPCR were performed using a Biorad iCycler and the SYBR green technology. Classical PCR primers were designed using the Primer3 software (http://frodo.wi.mit.edu/cgi-bin/primer3/primer3_www.cgi); primers for Q-rtPCR were designed using the Beacon Designer 6 software. Primers used for PCR, Q-rtPCR and EMSA experiments are listed in Table 1. All restriction endonucleases were purchased from Fermentas. T4 DNA ligase was obtained from Takara. The Pdx-1 cDNA was obtained by PCR amplification from reverse-transcribed total RNA from mouse pancreas and cloned in the unique Xho I site of the pcDNA3 vector, thus obtaining pcDNA3/Pdx-1; correctness of the insert was checked by automated sequencing. The pGL2-HK2-Luc construct used in the dual-luciferase reporter gene assay was kindly provided by prof. P. Pedersen [27].

*Electro Mobility Shift Assay (EMSA):* double stranded DNA probes were prepared by α-(32P)ATP labeling of oligonucleotides with Klenow DNA polimerase fragment followed by purification on G-25 columns (Amersham). Ten μg of nuclear protein extracts were incubated with 1 μg poly dI-dC, 150-300 fmol of radiolabelled specific double stranded probe, with or without a 100 fold excess of specific or unspecific unlabelled double stranded competitor for 30 minutes at RT. Reactions were resolved on 5% polyacrylamide gel in 0.5x TBE at 120 V for three hours, the gel dryed at 80 °C for 45 minutes; radioactive signal was detected by autoradiography exposing Kodak BioMax MS films for 12 to 48 hours at -80 °C. The putative PDX-1 binding site in the Hxk2 promoter was identified using the MatInspector on-line DNA sequence analysis software (www.genomatix.de).

*Antibodies and Western Blotting:* the goat anti-PDX-1 (A17) antibody (Santa Cruz, sc14664) was used both for western blotting and immunofluorescence. The mouse monoclonal anti-actin clone AC-40 antibody used for Western blotting was purchased from Sigma (cat. # A-4700). Western blotting and immunofluorescence were performed using standard procedures.

*Dual-luciferase reporter gene assay:* reporter gene assay for Hxk 2 promoter activity has been performed using the DUAL LUCIFERASE ASSAY SYSTEM from Promega; the reporter plasmid (pGL2-Hxk2-Luc) and the reference plasmid (pRL-Tk) were transfected at a ratio of 10:1 (1 μg total DNA). Protein extracts were prepared as already described, quantified, subsequently firefly and *Renilla* luciferase activity was measured in a manual luminometer setting the parameters as recommended by the kit manufacturer. The values obtained from *Renilla* luciferase activity were used to normalize the transfection efficiency of all samples.

*Measurement of glycogen synthesis:* confluent cells were washed five times with PBS and scraped in 500 μL PBS. One tenth of the cell suspension was saved and used to prepare total protein extract for quantification. The remaing 9/10 were spun and resuspended in 300 μL 20% KOH and solubilized for 2 hours. Tricloroacetic acid was added to 8% final concentration to deproteinize the extraxct, followed by neutralization by 2 M HCl. After adding 1 μg of glycogen to each sample, total glycogen was precipitated with ethanol (66% final concentration) for 1 hour at - 20° and pelleted by centrifugation. The pellet was dissolved in water and precipitated as above. Radioactivity was detected by liquid scintillation counting.

*Statistical analysis:* all data are presented as mean values of at least 3 independent experiments ± standard deviation (mean ± SD) or ± standard error (mean ± SEM).
